# Near Real-Time Surveillance of U.S. Norovirus Outbreaks by the Norovirus Sentinel Testing and Tracking Network — United States, August 2009–July 2015

**DOI:** 10.15585/mmwr.mm6607a1

**Published:** 2017-02-24

**Authors:** Minesh P. Shah, Mary E. Wikswo, Leslie Barclay, Anita Kambhampati, Kayoko Shioda, Umesh D. Parashar, Jan Vinjé, Aron J. Hall

**Affiliations:** ^1^Epidemic Intelligence Service, CDC; ^2^Division of Viral Diseases, National Center for Immunizations and Respiratory Diseases, CDC; ^3^Oak Ridge Institute for Science and Education, Oak Ridge Associated Universities.

Norovirus is the leading cause of endemic and epidemic acute gastroenteritis in the United States (*1*). New variant strains of norovirus GII.4 emerge every 2–4 years (*2*–*4*) and are often associated with increased disease and health care visits (*5*–*7*). Since 2009, CDC has obtained epidemiologic data on norovirus outbreaks from state health departments through the National Outbreak Reporting System (NORS) (*8*) and laboratory data through CaliciNet (*9*). NORS is a web-based platform for reporting waterborne, foodborne, and enteric disease outbreaks of all etiologies, including norovirus, to CDC. CaliciNet, a nationwide electronic surveillance system of local and state public health and regulatory agency laboratories, collects genetic sequences of norovirus strains associated with gastroenteritis outbreaks. Because these two independent reporting systems contain complementary data, integration of NORS and CaliciNet records could provide valuable public health information about norovirus outbreaks. However, reporting lags and inconsistent identification codes in NORS and CaliciNet records have been an obstacle to developing an integrated surveillance system.

In 2012, CDC launched Norovirus Sentinel Testing and Tracking (NoroSTAT), a collaborative network with selected state health departments that report specific epidemiologic and laboratory data on norovirus outbreaks to CDC via NORS and CaliciNet within 7 business days, and provide consistent identification codes for each outbreak (https://www.cdc.gov/norovirus/reporting/norostat/). The five states initially participating in NoroSTAT reduced reporting lag to NORS from a median of 22 to 2 days (p<0.001) and to CaliciNet from a median of 21 to 3 days (p<0.001). Nonparticipating states had no change in reporting lag to NORS, with a median of 26 days pre- and post-NoroSTAT implementation, and a reduction in reporting lag to CaliciNet from a median of 21 to 11 days (p<0.001). CaliciNet outbreaks that were linkable to NORS outbreaks increased from 86% to 95% (p<0.001) for NoroSTAT states, and from 29% to 33% (p = 0.016) for other states. NoroSTAT effectively integrates epidemiologic and laboratory surveillance data to provide near real-time monitoring of norovirus outbreak activity in the United States, thereby improving public health surveillance and guiding appropriate response.

Norovirus outbreak reports in NORS and CaliciNet from the five states that participated in the first 3 years of NoroSTAT (Minnesota, Ohio, Oregon, Tennessee, and Wisconsin) were compared with all other U.S. states, Washington, DC, and Puerto Rico. Only outbreaks reported with norovirus as the single confirmed or probable disease etiology were included. All transmission modes for norovirus (person-to-person, water, food, environment, and unknown) were included. The 3-year period after NoroSTAT introduction (August 2012–July 2015, “post-NoroSTAT”) was compared with the preceding 3-year period (August 2009–July 2012, “pre-NoroSTAT”).

Per capita reporting rates for each state were calculated by dividing the number of outbreaks reported to NORS and CaliciNet by U.S. Census intercensal yearly population estimates (*10*), and were expressed as outbreaks per 1,000,000 person-years. Report timeliness for NORS was assessed by determining the interval in days between the first report of an outbreak to a state health department and the date a NORS report was submitted. Report timeliness for CaliciNet was assessed as the difference in days between the date of receipt of a stool specimen at a public health laboratory and the date a CaliciNet report was submitted. The proportion of reports submitted within 7 business days was evaluated.

A complete NORS report was defined as a report that contained complete information for all fields required for NoroSTAT participation: date the first person in the outbreak became ill, primary transmission mode, exposure setting, and the number of ill persons. Linking ability was defined as the ability to link CaliciNet reports manually with a NORS report by user-submitted identification variables (automated linking was not possible during the period of analysis). The proportion of linked reports was assessed for NoroSTAT states and for other states. Norovirus genotypes reported to CaliciNet by NoroSTAT states and by other states were evaluated pre- and post-implementation of NoroSTAT. The primary transmission modes and outbreak settings reported to NORS by states participating in NoroSTAT were compared with other states and were similarly evaluated pre- and post-NoroSTAT implementation. CaliciNet-NORS linked reports were further analyzed to determine the distribution of reported transmission modes, outbreak settings, and norovirus genotypes from each reporting system. All analyses were completed using statistical software. Wilcoxon’s signed-rank test was used for numerical comparisons of reporting lag and outbreak size, and chi-square tests or Fisher’s exact tests were used for categorical comparisons, with a p-value <0.05 considered statistically significant.

The median outbreak reporting rates to NORS from NoroSTAT states before and after NoroSTAT introduction were similar (17.3 and 21.0 per 1,000,000 person-years, respectively) (Table 1). The median reporting rate to NORS from non-NoroSTAT states increased, from 3.0 per 1,000,000 person-years (pre-NoroSTAT) to 4.1 (post-NoroSTAT). The median reporting rate to CaliciNet from NoroSTAT states increased significantly from 4.9 (pre-NoroSTAT) to 9.0 (post-NoroSTAT). The median reporting rate to CaliciNet from non-NoroSTAT states was similar pre- (2.6) and post-NoroSTAT (2.1).

**TABLE 1:** Reporting indicators of norovirus outbreaks reported to the National Outbreak Reporting System (NORS) and CaliciNet — Norovirus Sentinel Testing and Tracking, United States,* August 2009–July 2015

Reporting indicators	NoroSTAT states (n = 5)			Other states (n = 47)		
	Pre-NoroSTAT	Post-NoroSTAT	p-value	Pre-NoroSTAT	Post-NoroSTAT	p-value
	Aug 2009–Jul 2012	Aug 2012–Jul 2015		Aug 2009–Jul 2012	Aug 2012–Jul 2015	
**Total NORS reports**	**1,357**	**1,981**	**—**	**2,843**	**3,738**	**—**
Median NORS reports per 1,000,000 p-y* (range)	17.3 (4.1–32.8)	21.0 (6.1–41.5)	0.16	3.0 (0.1–55.1)	4.1 (0.1–72.2)	0.045
Median reporting lag (days)	22	2	<0.001	26	26	0.29
No. (%) reported within 7 business days	359 (26)	1,888 (95)	<0.001	328 (12)	500 (13)	0.026
No. (%) with all required fields completed	1,183 (87)	1,979 (99.9)	<0.001	1,235 (43)	2,396 (64)	<0.001
**Total CaliciNet reports**	**657**	**1,077**	**—**	**1,885**	**2,174**	**—**
Median CaliciNet reports per 1,000,000 p-y (range)	4.9 (1.9–22.0)	9.0 (2.6–26.2)	0.036	2.6 (0.05–34.6)	2.1 (0.08–24.7)	0.63
Median reporting lag (days)	21	3	<0.001	21	11	<0.001
No. (%) reported within 7 business days	188 (29)	1,018 (95)	<0.001	196 (10)	651 (30)	<0.001
No. (%) linkable to NORS reports with reporter-supplied ID	564 (86)	1,027 (95)	<0.001	552 (29)	718 (33)	0.016

**Abbreviations:** NoroSTAT = Norovirus Sentinel Testing and Tracking; p-y = person-years.

*NoroSTAT-participating states during 2009–2015 were Minnesota, Ohio, Oregon, Tennessee, and Wisconsin. Washington, DC and Puerto Rico are included with other states.

The median reporting interval to NORS significantly declined in NoroSTAT states from 22 to 2 days and to CaliciNet from 21 to 3 days (Table 1). No change in reporting interval to NORS occurred in non-NoroSTAT states (median = 26 days pre- and post-NoroSTAT); however, there was a significant decline in the median reporting interval to CaliciNet from 21 to 11 days. The percentage of NORS reports submitted within 7 business days increased significantly, from 26% to 95% among NoroSTAT states, and increased marginally in non-NoroSTAT states (from 12% to 13%). The percentage of CaliciNet reports submitted within 7 business days increased from 29% to 95% among NoroSTAT states; a more modest, but still significant increase (10% to 30%) occurred among non-NoroSTAT states.

NORS reports with all NoroSTAT-required fields completed increased significantly, from 87% to 99.9% among NoroSTAT states, and from 43% to 64% in non-NoroSTAT states. The percentage of CaliciNet reports that were linkable to NORS reports also increased significantly among both NoroSTAT states (from 86% to 95%) and all other states (from 29% to 33%). Over the entire 6-year period, 2,861 CaliciNet reports linked to NORS reports across all states, providing more complete data on the outbreaks than either system alone. The NORS reports provided the transmission mode for all 1,106 (100%) linked CaliciNet records without an identified transmission mode, and the outbreak setting for 669 (60%) CaliciNet records without an identified setting. In addition, CaliciNet reports provided the genotype for all 719 linked NORS records without a reported genotype.

The genotypes of norovirus outbreaks reported to CaliciNet by NoroSTAT states were similar to those reported from non-NoroSTAT states (Table 2). In the pre-NoroSTAT period, GII.4 New Orleans was the dominant genotype in both NoroSTAT and non-NoroSTAT states, accounting for 54% and 65% of outbreaks in these states, respectively. The dominant genotype shifted to GII.4 Sydney in the post-NoroSTAT period in both NoroSTAT and non-NoroSTAT states, accounting for 61% and 68% of outbreaks, respectively. Similarly, the transmission modes and outbreak settings reported to NORS by NoroSTAT states were representative of national data, both pre- and post-NoroSTAT implementation (Table 2). Person-to-person transmission was the predominant transmission mode, and long-term care facilities were the most common outbreak setting.

**TABLE 2:** Reported genotype, transmission mode, and outbreak setting of norovirus outbreaks before and after implementation of NoroSTAT surveillance in five states* — United States, August 2009–July 2015

Variable reported	Pre-NoroSTAT (August 2009–July 2012)		Post-NoroSTAT (August 2012–July 2015)	
	NoroSTAT states No. (%)	Other states No. (%)	NoroSTAT states No. (%)	Other states No. (%)
Genotype^†^	657 (100)	1,885 (100)	1,077 (100)	2,174 (100)
GII.4 Den Haag 2006	39 (6)	121 (6)	4 (0.4)	5 (0.2)
GII.4 Osaka 2007	5 (0.8)	5 (0.3)	0	0
GII.4 New Orleans 2009	357 (54)	1,233 (65)	24 (2.2)	54 (2.5)
GII.4 Sydney 2012	4 (0.6)	22 (1.2)	657 (61)	1,474 (68)
Other GII	155 (23)	347 (18)	226 (21)	319 (15)
All GI	97 (15)	157 (8)	166 (15)	322 (15)
**Transmission mode^§^**	**1,357 (100)**	**2,843 (100)**	**1,981 (100)**	**3,738 (100)**
Person-to-person	958 (71)	2,191 (77)	1,456 (73)	2,952 (79)
Foodborne	236 (17)	515 (18)	296 (15)	550 (15)
Environmental	7 (0.5)	9 (0.3)	7 (0.4)	12 (0.3)
Unknown	156 (11)	128 (4)	222 (11)	224 (6)
**Outbreak setting^†^**	**995 (100)**	**1,129 (100)**	**1,674 (100)**	**2,330 (100)**
Long term care facility	818 (82)	856 (76)	1,183 (71)	1,728 (74)
Restaurant	19 (2)	18 (2)	59 (4)	59 (3)
Hospital/Other health care setting	41 (4)	89 (8)	69 (4)	116 (5)
Child care center	21 (2)	22 (2)	57 (3)	47 (2)
Other	96 (10)	144 (13)	306 (18)	380 (16)

**Abbreviation:** NoroSTAT = Norovirus Sentinel Testing and Tracking.

* NoroSTAT-participating states during 2009–2015 were Minnesota, Ohio, Oregon, Tennessee, and Wisconsin. Washington, DC and Puerto Rico are included with other states.

^†^ Norovirus genotypes as reported to CaliciNet.

^§^ Transmission modes and outbreak settings as reported to the National Outbreak Reporting System (NORS).

## Discussion

Substantial improvements in norovirus outbreak reporting, measured by the volume, timeliness, and completeness of epidemiologic and laboratory reports have been observed since the introduction of NoroSTAT in participating states, likely because of stringent reporting requirements and enhanced communication between epidemiologists and laboratorians in both state health departments and at CDC. NoroSTAT participating states are providing near real-time norovirus outbreak surveillance data to CDC, with 95% of NORS and CaliciNet reports submitted by these states within 7 business days. States not participating in the NoroSTAT network saw more modest improvements in report timeliness and completeness, likely because of general improvements in the report submission user interface, and increased engagement of state health departments and laboratories with annual meetings, workshops, and newsletters.

Ninety-five percent of CaliciNet reports submitted by NoroSTAT states were linked to NORS reports, fostering better integration and coordination of epidemiologic and laboratory data. These linked reports provide more complete and accurate reporting of norovirus outbreaks, and their value is illustrated by the large proportion of reports for which data from one system supplement those from the other. Report linkages were completed manually and retrospectively during the period of this analysis; automated, prospective linking is currently being implemented.

The findings in this report are subject to at least two limitations. First, both NORS and CaliciNet collect data on norovirus outbreaks; because norovirus outbreaks are defined as two or more cases of illness with a common exposure, these results might not be generalizable to endemic norovirus illnesses. Second, variations in reporting practices among both NoroSTAT and non-NoroSTAT states can affect the quality and internal comparability of surveillance data.

Despite these limitations, the NoroSTAT network was shown to be valuable in early identification and better characterization of norovirus outbreaks across the country. Near real-time surveillance data from NoroSTAT improved public health response and preparedness when the GII.4 Sydney variant emerged during 2012–2013. Early reporting of data from NoroSTAT states allowed a timely assessment showing no increase in norovirus outbreak activity in the United States associated with emergence of the GII.4 Sydney variant, in contrast to data from other countries (*4*). Similar strain-specific attribution analyses can be useful to rapidly detect the impact of the emergence of novel norovirus strains every few years.

The NoroSTAT network has built upon the initial success demonstrated by the first five states, expanding to seven states in August 2015 and to nine states in August 2016. The more rapid, complete, and integrated reporting by NoroSTAT-participating states demonstrates a key advancement in norovirus outbreak surveillance, providing near real-time monitoring of norovirus outbreak activity and emerging new strains.

**Summary**

**What is already known about this topic?**

Norovirus is the most common cause of acute gastroenteritis in the United States. Norovirus outbreaks are reported to CDC by state and territorial health departments. Reporting lags and incomplete reporting have been limitations to norovirus outbreak surveillance systems.

**What is added by this report?**

The initial five sentinel states that participated in the NoroSTAT network (Minnesota, Ohio, Oregon, Tennessee, and Wisconsin) during the first 3 years reduced the median reporting interval from 22 days to 2 days for epidemiologic data, and from 21 days to 3 days for laboratory data. These states also had more complete reports that better linked epidemiologic and laboratory data.

**What are the implications for public health practice?**

The NoroSTAT network provides near real-time surveillance of norovirus outbreak activity and emerging new strains. Data collected by NoroSTAT-participating states are representative of national trends and can help inform public health response.
